# Basket of options: Unpacking the concept

**DOI:** 10.1177/00307270211019427

**Published:** 2021-07-07

**Authors:** E Ronner, J Sumberg, D Glover, KKE Descheemaeker, CJM Almekinders, BIG Haussmann, TW Kuyper, H Posthumus, P Ebanyat, KE Giller

**Affiliations:** 1 Plant Production Systems, 8225 Wageningen University, Wageningen, The Netherlands; 2 Institute of Development Studies, Brighton, UK; 3 Knowledge, Technology and Innovation, 8225 Wageningen University, Wageningen, The Netherlands; 4 Institute of Plant Breeding, Seed Science and Population Genetics, 26558 University of Hohenheim, Stuttgart, Germany; 5 Soil Biology, 8225 Wageningen University, Wageningen, The Netherlands; 6 1226 KIT Royal Tropical Institute, Amsterdam, The Netherlands; 7 School of Agricultural Sciences, 58588 Makerere University, Kampala, Uganda

**Keywords:** Agricultural innovation, option x context, agricultural extension, co-learning, smallholder agriculture

## Abstract

How to stimulate technological change to enhance agricultural productivity and reduce poverty remains an area of vigorous debate. In the face of heterogeneity among farm households and rural areas, one proposition is to offer potential users a ‘basket of options’ – a range of agricultural technologies from which potential users may select the ones that are best suited to their specific circumstances. While the idea of a basket of options is now generally accepted, it has attracted little critical attention. In this paper, we reflect on outstanding questions: the appropriate dimensions of a basket, its contents and how they are identified, and how a basket might be presented. We conceive a basket of options in terms of its depth (number of options related to a problem or opportunity) and breadth (the number of different problems or opportunities addressed). The dimensions of a basket should reflect the framing of the problem or opportunity at hand and the objective in offering the basket. We recognise that increasing the number of options leads to a trade-off by decreasing the fraction of those options that are relevant to an individual user. Farmers might try out, adapt or use one or more of the options in a basket, possibly leading to a process of technological change. We emphasise that the selection (or not) of specific options from the basket, and potential adaptation of the options, provide important opportunities for learning. Baskets of options can therefore be understood as important boundary concepts that invite critical engagement, comparison and discussion. Significant knowledge gaps remain, however, about the best ways to present the basket and to guide potential users to select the options that are most relevant to them.

## Introduction

Since the 1960s, agricultural research and extension in developing countries have been re-orienting from on-station to on-farm activities, from favourable to risk-prone environments, and from top-down and linear to client-oriented, participatory and adaptive approaches (Bingen and Gibbon, 2012; Chambers and Ghildyal, 1985; Leeuwis and Van den Ban, 2004). Yet, particularly in sub-Saharan Africa (SSA), there is a continuing sense of disappointment with the rate of technological change on smallholder farms: productivity remains low, while rural poverty rates remain high (Thurlow et al., 2019). There is an urgent need for new ways to conceptualise and study processes of technological change (Glover et al., 2019), and for new tools and methods to support such processes. Moreover, there is increasing pressure to extend site-specific findings obtained through intensive interactions with a limited number of individuals into scalable initiatives (De Roo et al., 2019; Glover et al., 2016; Woltering et al., 2019).

In the face of heterogeneity among farm households and rural areas, the concepts of choice and agency have been important to the evolution of more adaptive and user-oriented approaches to agricultural research and extension. Framed as presenting farmers with a ‘basket of options’, a ‘basket of choices’, a ‘menu of options’ or ‘relevant sets of options’, the idea is that farmers should be able to choose agricultural technologies that are best suited to their specific socio-economic and agro-ecological circumstances and their aspirations.

This approach is attractive for two key reasons. It recognises the knowledge, experience and agency of farmers to decide what is most suitable for their specific situations; and it reduces the burden for research and extension to develop specific recommendations that address thousands of possible situations. But while the idea of working with a basket of options is now generally accepted (Descheemaeker et al., 2019; Giller et al., 2011; Woomer, 2007), and generates little controversy, it has attracted little critical attention. A number of important questions remain, and in this paper we address several of them: How might a basket of options be characterised? How many different options might the basket contain, or how many different problems or opportunities might it address? How narrow or wide a population of potential users might a basket of options target? How are the options in a basket developed and presented? What is the role of farmer feedback on the options? And finally, how might the notion of a basket of options inform approaches to scaling?

In an effort to establish a more solid foundation for the basket of options concept, we begin by placing it within the general context of agricultural research and extension and provide a short history of its evolution. We then reflect critically on the questions identified above. Finally, we discuss the implications of this reflection for agricultural research and extension. While we draw primarily on examples of technological change associated with crop production in smallholder agriculture, the discussion is relevant to all other farm production enterprises and broader livelihood strategies.

## Recommendations, options and baskets

The configuration of relationships between agricultural research, extension, and farming practice has long been a topic of debate, including the relative importance and roles of fundamental research, applied research, extension and farmer involvement. Providing information, advice and recommendations to farmers is the bread and butter of agricultural extension (Leeuwis and Van den Ban, 2004). From the early days in the USA, crop variety testing was a key extension activity (e.g. Pellack and Karlen, 2017), with information about the characteristics and performance of different cultivars being made available to farmers. This suggests that, at least in some times and places, there is nothing new or radical in the idea of agricultural extension providing information about options or alternatives. However, the dominant narrative is that agricultural extension in sub-Saharan Africa (SSA) was different from what was seen in North America, Europe and some other developed countries. Specifically, the conventional view is that, from colonial times until the 1980s, extension essentially channelled a one-way flow of technical ‘recommendations’ from research to farmers (Klerkx et al., 2012). The thrust was to ‘modernise’ crop production through, for example, line planting, better spacing, timely weeding and the optimal use of fertiliser and improved varieties. From this perspective, underpinned by an assumption that farmers were ignorant, or at least highly resistant to change, extension relied on relatively simple blanket recommendations, and measured success in terms of the ‘adoption’ of technologies being promoted. These *dirigiste* approaches to extension were often rationalised in the light of, for example, the critical role that export crops like cocoa, groundnut and cotton played in some colonial and post-independence economies; the assumed need to control crises of deforestation and soil erosion (Tiffen et al., 1994); plans to produce export crops on large-scale irrigation schemes (Baldwin, 1957); or the desire to ‘settle’ rural people through the introduction of mixed crop-livestock farming (Sumberg, 1998; Wolmer and Scoones, 2000).

As African countries gained their independence, food crops received more attention from agricultural research. But advances in Asian food crop productivity associated with the Green Revolution highlighted the absence of a corresponding degree of technical progress among smallholders in SSA. This lack of progress was partly attributed to top-down extension approaches that promoted technologies based on blanket recommendations that were not suitable to the majority of smallholder farmers. New approaches to agricultural research and extension emerged in the 1980s, including the farming systems research movement (Collinson, 2000). To get around the problem of blanket recommendations, the concept of ‘recommendation domains’ was introduced, followed by the idea of a ‘farmer first’ approach (Chambers et al., 1989) emphasising farmers’ own experimentation (Sumberg and Okali, 1997). The idea was that farmers’ perspectives should become increasingly important, as (in the eyes of research and extension staff) they transitioned from passive recipients of technology to clients, collaborators, stakeholders and, in some cases, funders of research. More emphasis was placed on farmers’ criteria in the identification of problems and evaluation of technology (Byerlee, 1987).

It is in this context that the notion of a basket (or menu) of options (or choices) emerged. The starting point was probably the 1987 conference on ‘Farmers and Agricultural Research: Complementary Methods’, organised by Robert Chambers. In Chapter 4.4 of the resulting *Farmer First* book (Chambers et al., 1989), Chambers challenged the training and values that reproduce the ‘normal professionalism’ of agricultural research and extension personnel. He argued that this normal professionalism underpinned the transfer-of-technology (TOT) mode, which he characterised as ‘scientists deciding research priorities, generating technology and passing it on to extension agents to transfer to farmers’ (pp. 181–182). Chambers contrasted TOT with the ‘farmer first approach’, in which outsiders would transfer principles, methods and a ‘basket of choices’ to farmers, whereas under TOT they would transfer precepts, messages and a ‘package of practices’. With farmer first, the ‘menu’ was supposed to be ‘à la carte’, while under TOT it was ‘fixed’. In a paper published in the interval between the 1987 conference and the appearance of the *Farmer First* book in 1989, Chambers (1988) wrote that:

…this transfer of technology approach does not work very well with the…complex, diverse and risky farming systems [of poor farmers and resource-poor areas]. Instead many pioneering workers have now shown that a holistic approach is better, allowing everything in a farming system to be potentially relevant. For this, diagnosis is best done by farmers themselves, with scientists or extensionists in a support role. This is a major reversal. **The menu which comes out is not fixed, table d’hôte, but à la carte, not a package of practices but a basket of choices. Farmers can select from a wider range of technology, enhancing their adaptability. The role of outsiders is to learn from and with farmers, and to give them choices, while farmers choose from the basket and conduct their own trials and experiments**. (p. 53, emphasis added)

Others, including Nelson (1988) were quick to pick up on the idea of a basket or menu of options. Chambers himself referred to it repeatedly in subsequent years (Chambers, 1990; 1991a; 1991b; 1991c; 1992; 1993). Interest in baskets and menus continued into the early 2000s (e.g. Malama and Kondowe, 2003; Vanlauwe et al., 2003), with Snapp et al. (2002) referring to a ‘range of options’, and Kebbeh and Miezan (2003) to a ‘crop management technology basket’, while Bonny et al. (2005) talked of a ‘basket of scientifically proven options’. Towards the end of the decade, Woomer (2007) was suggesting that it was already ‘conventional wisdom’ that ‘food security in Africa will be achieved by presenting smallholder farmers with a “basket” of crop and land management options from which they may choose the practices that best suit their site-specific needs and socio-economic conditions’ (p. 881). But while the image of a basket had taken root, Woomer critiqued continuing adherence to ‘failing “top-down” models of dissemination’ in which farmers are ‘at best’ minimally involved in technology development and different options are formulated on ideological principles and developed in isolation from one another.

Baskets of options have featured in a range of recent work, including papers by Giller et al. (2011), Falconnier et al. (2017), Ronner (2018), and Descheemaeker et al. (2019), who couple them with iterative, co-learning cycles. The recognition of the importance of tailoring options to local contexts is reflected in the shift from more general ‘best-bet’ options to ‘best fits’ – options that are assessed for their suitability to fit within a particular context or niche (Giller et al., 2011; Ojiem et al., 2006). Following this line of thinking, Coe et al. (2014), Nelson and Coe (2014), Nelson et al. (2016) and Sinclair and Coe (2019) focus on matching ‘locally adapted options’ and ‘relevant sets of options’ to different contexts.

## Critical reflections on the ‘basket of options’ concept

### Baskets

Imagine two restaurants. In each establishment, a customer enters and asks for the menu: in the first she is handed a single sheet of paper that contains two choices: ‘spaghetti bolognaise’ or ‘spaghetti napolitana’. In the second, she is given a document several pages long, with multiple entries (each described in wondrous detail) under a variety of headings (Antipasti, Primi, Secondi, Contorni, Insalata, Formaggi e frutta, Dolce, Caffe). In both cases she was given a menu; the simple point is that all menus – all baskets of options – are not the same.

**Figure 1.**
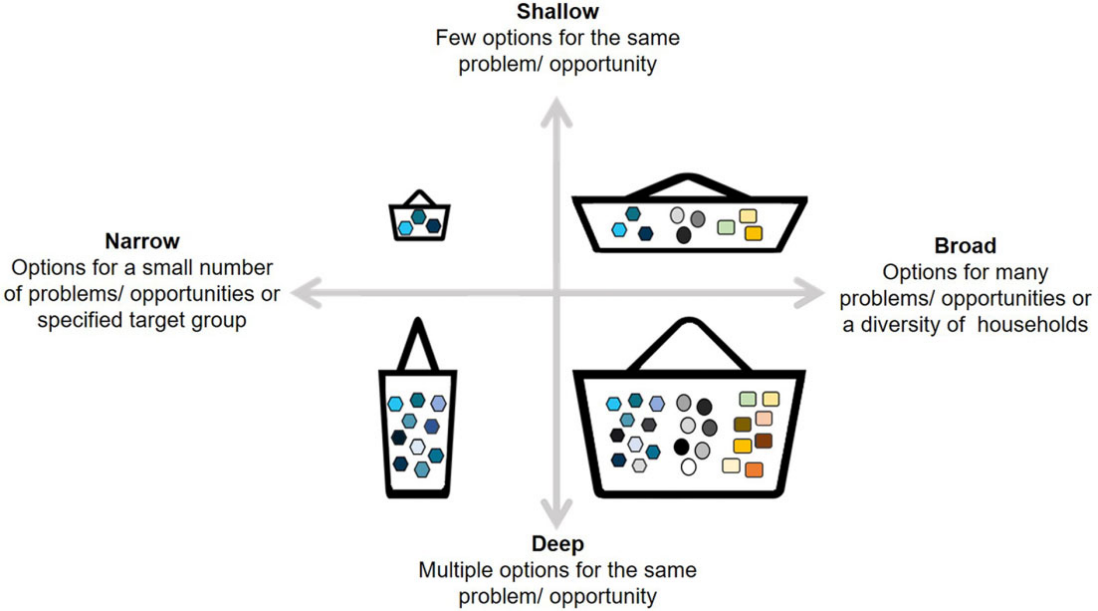
Stylised baskets of options, where colours within the same shapes represent different options for the same problem/opportunity, while different shapes represent different problems/opportunities being addressed.

A notional basket of options can be understood in terms of its *depth* and *breadth* (Figure 1). The depth of the basket refers to the number of options it contains relating to a particular problem or opportunity, while the breadth refers to the number of different problems or opportunities which the options in the basket seek to address. Table 1 provides an agricultural illustration based on Ronner et al. (2019). As with the restaurant example, it is clear that the six baskets of options depicted in the table are quite different. As such, they present both the basket developers and potential users with distinct challenges and considerations.

**Table 1.** Examples of baskets of options varying by depth (number of options for a particular problem/ opportunity) and breadth (number of problems or opportunities), loosely based on Ronner et al. (2019).

		**Depth of basket**	
		**Shallow**	**Deep**
**Breadth of basket**	**Narrow**	2 climbing bean varieties	10 climbing bean varieties
	**Broad**	2 climbing bean varieties + 1 fertiliser regime + 1 staking method 1 bean storage option + 1 collective marketing model	10 climbing bean varieties + 3 fertiliser regimes + 3 staking methods 3 bean storage options + 1 collective marketing model
	**Very broad**	2 climbing bean varieties + 1 fertiliser regime + 1 staking method 1 bean storage option + 1 collective marketing model + 2 bush bean varieties + 2 maize varieties	10 climbing bean varieties + 3 fertiliser regimes + 3 staking methods 3 bean storage options + 1 collective marketing model + 10 bush bean varieties + 10 maize varieties

First and foremost, the depth and breadth of a basket developed in a particular case should reflect 1) the framing of the problem or opportunity at hand, and 2) the objective in compiling and offering the basket. As agricultural research and extension addresses a broad spectrum of problems and opportunities, interacts with different types of farmers in a diversity of contexts and does so for various reasons, we can expect baskets to vary significantly in depth and breadth. For example, a problem framing that highlights the limited genetic base underpinning maize production in an already vibrant maize production area might lead to the creation of a narrow and relatively deep basket that contains only new maize varieties. In contrast, a basket constructed in response to a problem framing that highlights a generally low level of productivity and poor grain quality might be broad and shallow, including new genetic material, new crop management practices and new storage methods.

As a general rule it is reasonable to expect that the more heterogeneous the agro-ecological setting, institutional context and/or population of potential users, the deeper and/or broader the basket would be – i.e. the more options it would contain (Figure 1). However, there is a clear trade-off at play, in that a large number of options within a basket may increase the search costs to a potential user. Options that are (or appear to be) irrelevant may become a distraction: a basket with too many options may give rise to information overload (Roetzel, 2019). In contrast, a narrower and more targeted basket would increase, on the researchers’ side, the proportional costs per user of creating the basket, as it requires a more detailed understanding of local conditions and farmers’ constraints and aspirations. Over time, the knowledge about the objectives and needs of the potential users and the performance and reliability of the options under local conditions would improve, so that the basket could be reshaped to an appropriate size to manage this trade-off. A deep and broad basket could also be subdivided into several narrow and shallow baskets, which might then be more effectively targeted to a particular agro-ecological niche or group of farmers. A key matter of judgement is: when is it better to design a broad and/or deep basket, instead of a narrow and/or shallow one?

In terms of objectives, an extension or development programme might be interested in providing farmers with a range of new options, which it believes will address important problems. In contrast, and perhaps beyond Chambers’ original thinking, a researcher might be interested in using a basket to obtain feedback on a set of options that are still under development, to study farmer’s preferences among or adaptations to the options, or to narrow down and refine the options to be included in a subsequent scaling programme. Farmers might be interested in exploring a basket of options that can help them meet multiple objectives. Farmers’ objectives might differ from those that research and extension professionals typically have in mind, for instance, maximising the productivity of labour rather than land, increasing resilience instead of maximising yield, or meeting the needs of domestic consumption rather than the market. The range of objectives conceived by different stakeholders suggest the scope for baskets of different dimensions, containing different types of options.

### Options

Options are alternatives. By placing two or more options in a basket, the basket developer is essentially saying to a potential user ‘here are some options that *might* help you address problem X’. In this sense, an option is what Glover et al. (2019) refer to as a ‘proposition’, which ‘conjures up the possibility of an alternative way of working or making to achieve new or different outcomes’ (p. 6). The suggestion is that every proposition includes (1) some biophysical resources, such as seeds, tools, equipment, machines, energy and built infrastructure, (2) methods, techniques and/or practices and a set of more or less specific instructions, recommendations, guidelines or protocols, and (3) a proposed ‘mode of engagement’ that embodies assumptions or suggestions about the motivations and capabilities of the farmers who will most likely engage with the proposition.

A proposition (‘you might try this’) is not the same as a recommendation (‘we recommend that you do it this way’). The intention behind a recommendation is that the person to whom it is made will react by implementing it as given. In contrast, the intention behind a proposition is to provide an opportunity to respond (or not) to something new, with the explicit understanding that people may respond in various ways, some of which will not have been anticipated or intended by those who developed or presented the proposition. A basket of options can be considered as a set of propositions. An individual who is exposed to the basket might decide to engage with – to try out, adapt or adopt – one or more of these options, depending on her/his objectives, aspirations, capabilities, and so on.

Options might range all the way from highly specified technologies that leave little room for local adaptation (e.g. a livestock vaccine), to more generic ideas or principles (e.g. keep the soil covered). Where an option sits along this continuum will help determine how much room and need there is for potential users to adapt it to their own agro-ecological, socio-economic and personal circumstances; and to what extent researchers may need to be involved in the adaptation process – less for highly specified technologies, more for relatively complex or knowledge intensive ‘systems’ technologies (cf. Descheemaeker et al., 2019; Marinus et al., 2021; Reece and Sumberg, 2003).

Screening the options to be included in a basket should be guided by the problem framing of and objective for developing the basket, as well as an assessment of the relevant parameters of local production systems, the biophysical, socio-economic and institutional conditions, and the cultural context. In some cases, the identification of options might be done by researchers using relevant literature, their own experience or experimental data, while in other cases the identification might require intensive engagement with and input by or feedback from potential users (Sumberg et al., 2003). The process of identifying options may be informed by the extensive experience and literatures dealing with agricultural technology development and evaluation – including on-station, on-farm, formal, informal, researcher-managed, farmer-managed, farmer-driven and participatory (Bellon, 2001; Defoer, 2002; Nelson et al., 2016). The wider literatures on ‘new product development’, Science and Technology Studies, co-design or user-centred design also offer important insights on how user involvement may enhance the relevance of identified options (Meynard et al., 2012; Pinch and Bijker, 1984; Sumberg et al., 2013). Any plan to include farmers and other stakeholders in the design, assessment and/or selection of options to be included in a basket must be based on careful consideration of three questions: What is the objective of their involvement? Who should be involved? At what point(s) in the process will their involvement be most useful?

### Presenting a basket

Careful consideration must be given to how the options in the basket are presented (e.g. through what Glover et al. (2019) called ‘encounters’). The way this is done must be appropriate to the objective, the type of basket (its depth and breadth), and the specific options contained within it. The nature and quality of the encounter through which farmers are introduced to a basket of options will influence both how the basket and its contents are perceived, and what happens next. Proposing an option can be seen as a kind of nudge (Thaler and Sunstein, 2008), but rather than nudging towards a specific option, it is the broader behaviour of trying that is being encouraged.

An important part of any encounter is a presentation of the information that accompanies each option and that will help potential users to assess their interest in an option. How this is done will likely depend on the combination of problem framing and objective, and the nature of the options in the basket. For example, the information accompanying the presentation of a new crop variety or pesticide would likely be quite different from that accompanying a set of broad principles. For well-established options the information may largely be known beforehand, for other options it will have to be derived from and validated in farmers’ try-outs and evaluations. A question will likely also arise around whether the options should be presented as a set of relatively ‘fixed’ practices with specific instructions for their use, or as a more flexible set of tools, principles and concepts which farmers are encouraged to try, adapt and tailor to their own situation. Information may also be needed on certain prerequisites, or the expected consequences of using an option (e.g. if plant density increases, the risk of drought stress may also increase). From the perspective of those who designed or identified the options within a broad basket, there may be a preferred sequence of application (c.f. Integrated Soil Fertility Management, Vanlauwe et al., 2010), or a preferred combination of application (e.g. mulch and zero till in Conservation Agriculture) and these considerations will need to be discussed as well.

Simple heuristic tools might have a role in guiding potential users through the options in a basket. By prompting reflection on questions like ‘what’s my situation?’ and ‘what might work for me?’ (Glover, 2014), these tools could help focus attention on the options that are most likely to be of interest. Some examples include the ‘option-by-context’ matrix (Ronner et al., 2019; Sinclair and Coe, 2019), the Stepwise tool (Jassogne et al., 2017) or a decision tree (Okali et al., 1994).

### Learning from baskets

The motivation for and objective of setting out a basket of options will determine the most appropriate strategies for observation, evaluation, feedback and learning. These strategies might range from a simple and light touch (e.g. with a large-scale extension programme), to much more involved iterative co-learning cycles (Falconnier et al., 2017; Prost et al., 2018; Ronner et al., 2019). If managed with care, co-learning cycles could help to validate, refine or improve one or more options in the original basket, to re-structure the basket in terms of its depth and/or breadth, to change the nature of the encounter through which the basket is introduced, or to completely re-orient the basket (Marinus et al., 2021). To balance the need to gather context-specific information that makes the basket and the options locally relevant with the time and resources invested, there is a need for innovative methods and tools to bring farmer feedback and assessment fully into these learning processes, in ways that are both effective and efficient. Largely, this comes back to the shift in responsibilities and relationships between farmers, extensionists and researchers that Chambers (1988) already referred to. Researchers and extensionists would support farmers to conduct their own, simplified experiments, to gather meaningful data from these (e.g. through ICT), and to place the results in a wider context. Examples of such innovative approaches described in literature are Farmer Research Networks (Nelson et al., 2016) or triadic comparisons of technologies (tricot) (Van Etten et al., 2019).

## Implications for agricultural research and extension

### Baskets can help change the conversation

Rural people already navigate amongst various options and alternatives. They might farm full-time or part-time, grow multiple crops, encounter new technologies through an extension officer, farmer group or on a neighbour’s farm, and decide to try these out for one or more seasons. The literature on farmers’ experimentation demonstrates that farmers try out, compare and adapt different tools, techniques and methods as a normal part of a farming repertoire (Glover, 2018; Hockett and Richardson, 2016; Misiko and Tittonell, 2011; Sumberg and Okali, 1997). In principle then, moving from a single recommendation to a basket of options could enable conversations that are better grounded in farmers’ realities (cf. Almekinders et al., 2019; Mausch et al., 2021). In that sense, in addition to making new options available to potential users, baskets of options can serve as important boundary concepts that invite critical engagement, comparison and discussion between farmers, extension officers and researchers.

Critical engagement with the basket can be helped or hindered by the nature of the options (a highly specified option versus a generic principle), the way they are designed (with or without user involvement) and communicated, and the nature of the encounter through which they are introduced (as a one-time event or a longer-term participatory process). And as argued above, much depends on the problem framing and the objective in creating and introducing the basket. The point is that in order to realise the potential advantage of a basket of options approach, much more is required than simply generating and presenting sets of options. The inclusiveness of the process of defining the basket and the type of engagement with potential users will also influence the potential advantage of the approach and the perceived relevance of the basket to potential users, next to the nature of the options themselves (Almekinders et al., 2019).

### Baskets and scaling

The challenge of scaling in agricultural development processes is complex, multifaceted and contested (De Roo et al., 2019; Linn, 2012; Makate, 2019; Seifu et al., 2020). At first sight, a basket of options approach would appear to have potential to facilitate scaling. Especially when one considers scaling the approach itself rather than the specific options within the basket. Some challenges remain, however.

First, we have already mentioned the potential trade-off between using a basket as a way to provide diverse options to a large number of potential users across a variety of contexts, and the increased likelihood that many of these options will be less relevant for any given user. This generates the dilemma of choosing between baskets that are deep vs. shallow, and narrow vs. broad. It also calls for a systematic assessment of the relevant context variables for the options in the basket – some options may be applicable in a diversity of contexts, and hence may be more easily scaled than options that only perform well in a specific context (Nelson et al., 2016).

Second, a narrow basket for targeted scaling requires that the population of potential users is well-known, and that the characteristics of that population are relatively stable over time. A basket of drought-tolerant maize varieties will likely be of interest to people in arid areas. However, when a target population is defined by characteristics that may change within a season or over a short time-span (such as capital or labour availability, livestock or asset ownership; cf. Fraval et al., 2019; Hammond et al., 2020; Ronner et al., 2018), the relevance of the options in the basket may have a limited lifetime. In these situations, broader baskets combined with heuristic tools may be desirable.

Third, in addition to a potentially viable technological proposition, successful scaling of an option is likely to require additional changes in knowledge, incentives, markets, supply chains, organisational structures, coordination mechanisms and/or infrastructure (Kuehne et al., 2017; Schut et al., 2016; Woltering et al., 2019). This suggests that the successful use of baskets of options in scaling will need a broader understanding of these requirements, as well as the establishment of relevant partnerships to create and sustain access to the options in the basket.

Finally, a basket of options approach may have implications for the way research and development interventions are organised and how their success is measured. Many interventions are designed around specific crops, often founded in organisational expertise and mandate. If the organisation’s success is measured in the number of farmers adopting a new crop variety, a more diversified basket with options for multiple crops would limit their potential success. Hence, a reconsideration of the incentives for the organisations offering a basket, with a better connection to farmers’ preferences and aspirations, may help designing more meaningful rural development interventions (Almekinders et al., 2019; Mausch et al., 2021), but would also require reconsidering measures of success (Glover et al., 2016; Woltering et al., 2019).

## Conclusions

Key advantages of a basket of options approach are the potential to accommodate diversity, the recognition that the eventual use of an option may be quite different from what researchers had initially envisaged, and the potential it provides to start conversations about farmers’ constraints, objectives and imagined futures. A clear problem framing and objective will set the stage for the design of baskets of options that balance increased diversity with an appropriate relevance of options. The notion of a basket of options provides a useful boundary concept for framing agricultural research and extension efforts that seek to support farmers’ on-going efforts to try out and adapt new agricultural technologies to their own situations.

In this paper, we have addressed a serious gap in understanding how baskets of options can be developed, presented and used most effectively. However, there remains much scope to develop the ways in which baskets can be encountered; to take better account of local contexts, social norms, technological characteristic and so on. In relation to debates about scaling of technologies, there is need for reflection on the relevant context variables that help to determine successful scaling of options in a basket. Finally, there are significant knowledge gaps concerning the best way to communicate the relevant information needed to guide potential users through the basket, so that they can identify the (combination of) options that are most relevant and useful to them, and that may eventually lead to a process of technological change.
